# 1,3-Benzothia­zole-2(3*H*)-selone

**DOI:** 10.1107/S1600536811043339

**Published:** 2011-10-29

**Authors:** Gunay Z. Mammadova, Zhanna V. Matsulevich, Vladimir K. Osmanov, Alexander V. Borisov, Victor N. Khrustalev

**Affiliations:** a Baku State University, Z. Khalilov Street 23, Baku AZ-1148, Azerbaijan; b R. E. Alekseev Nizhny Novgorod State Technical University, 24 Minin Street, Nizhny Novgorod 603950, Russian Federation; c X-ray Structural Centre, A. N. Nesmeyanov Institute of Organoelement Compounds, Russian Academy of Sciences, 28 Vavilov Street, Moscow 119991, Russian Federation

## Abstract

The title compound, C_7_H_5_NSSe, is the product of the reaction of 2-chloro­benzothia­zole with sodium hydro­selenide. The mol­ecule is almost planar (r.m.s. deviation = 0.018 Å) owing to the presence of the long chain of conjugated bonds (Se=C—N—C=C—C=C—C=C). The geometrical parameters correspond well to those of the analog *N*-methyl­benzothia­zole-2(3*H*)-selone, demonstrating that the S atom does not take a significant role in the electron delocalization within the mol­ecule. In the crystal, mol­ecules form centrosymmetric dimers by means of inter­molecular N—H⋯Se hydrogen bonds. The dimers have a nonplanar ladder-like structure. Furthermore, the dimers are linked into ribbons propagating in [010] by weak attractive Se⋯S [3.7593 (4) Å] inter­actions.

## Related literature

For selones as potential anti­thyroid drugs, see: Taurog *et al.* (1994 ▶); Roy & Mugesh (2005 ▶, 2006 ▶); Roy *et al.* (2007 ▶, 2011 ▶). For 2,3-dihydro-1,3-benzothia­zolo-2-selone synthesis, see: Warner (1963 ▶); Shibata & Mitsunobu (1992 ▶). For related compounds, see: Guziec & Guziec (1994 ▶); Husebye *et al.* (1997 ▶); Landry *et al.* (2006 ▶); Nakanishi *et al.* (2008 ▶).
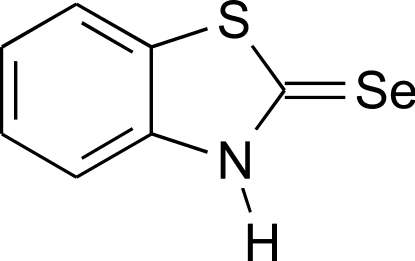


## Experimental

### 

#### Crystal data

C_7_H_5_NSSe
*M* *_r_* = 214.15
Monoclinic, 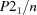

*a* = 8.0420 (4) Å
*b* = 6.0818 (3) Å
*c* = 15.1836 (7) Å
β = 101.195 (1)°
*V* = 728.50 (6) Å^3^
*Z* = 4
Mo *K*α radiation
μ = 5.35 mm^−1^
*T* = 100 K
0.30 × 0.21 × 0.18 mm

#### Data collection

Bruker SMART 1K CCD diffractometer
Absorption correction: multi-scan (*SADABS*; Sheldrick, 1998 ▶) *T*_min_ = 0.297, *T*_max_ = 0.446
8129 measured reflections
2108 independent reflections
2042 reflections with *I* > 2σ(*I*)
*R*_int_ = 0.020

#### Refinement

*R*[*F* ^2^ > 2σ(*F* ^2^)] = 0.016
*wR*(*F* ^2^) = 0.041
*S* = 1.00
2108 reflections
91 parameters
H-atom parameters constrained
Δρ_max_ = 0.44 e Å^−3^
Δρ_min_ = −0.29 e Å^−3^

### 

Data collection: *SMART* (Bruker, 1998 ▶); cell refinement: *SAINT* (Bruker, 1998 ▶); data reduction: *SAINT*; program(s) used to solve structure: *SHELXTL* (Sheldrick, 2008 ▶); program(s) used to refine structure: *SHELXTL*; molecular graphics: *SHELXTL*; software used to prepare material for publication: *SHELXTL*.

## Supplementary Material

Crystal structure: contains datablock(s) global, I. DOI: 10.1107/S1600536811043339/rk2309sup1.cif

Structure factors: contains datablock(s) I. DOI: 10.1107/S1600536811043339/rk2309Isup2.hkl

Supplementary material file. DOI: 10.1107/S1600536811043339/rk2309Isup3.cml

Additional supplementary materials:  crystallographic information; 3D view; checkCIF report

## Figures and Tables

**Table 1:** Hydrogen-bond geometry (Å, °)

*D*—H⋯*A*	*D*—H	H⋯*A*	*D*⋯*A*	*D*—H⋯*A*
N3—H3⋯Se1^i^	0.88	2.56	3.4165 (10)	163

Symmetry code: (i) 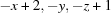.

**Fig. 1.**
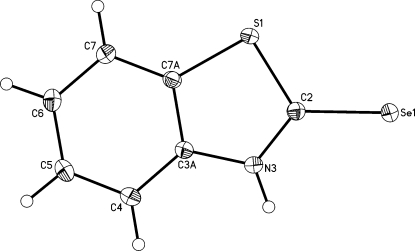
Molecular structure of title compound with the atom numbering scheme. Displacement ellipsoids are shown at the 50% probability level. The H atoms are presented as a small cyrcles of arbitrary radius.

**Fig. 2.**
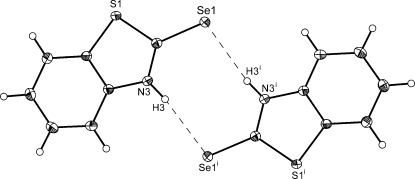
The centrosymmetrical dimers in crystal structure of I. Dashed lines indicate the intermolecular hydrogen bonds N3–H3···Se1i. Symmetry code: (i) -x+2, -y, -z+1.

**Fig. 3.**
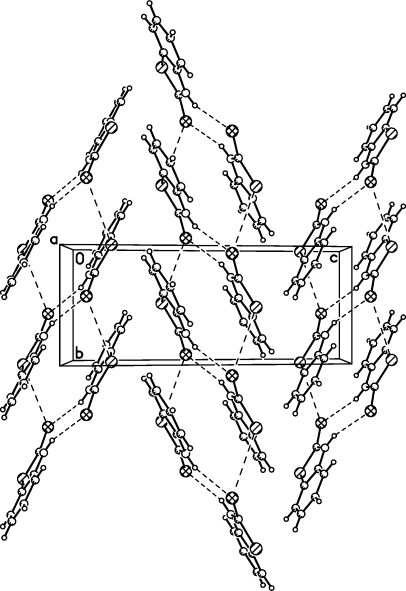
Crystal packing of dimers of I. Dashed lines indicate the intermolecular N–H···Se hydrogen bonding and weak attractive Se···S interactions.

**Fig. 4.**
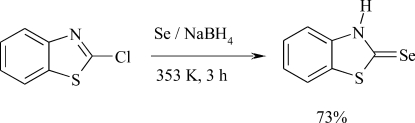
Reaction of 2-chlorobenzothiazole with sodium hydroselenide.

## supplementary crystallographic information

### Comment

In the last years, the selone derivatives have attracted considerable attention owing to their antithyroid properties (Taurog *et al.*, 1994; Roy & Mugesh, 2005, 2006; Roy *et al.*, 2007, 2011) as well as selone-selenol tautomerism (Guziec & Guziec, 1994; Husebye *et al.*, 1997; Landry *et al.*, 2006).

This article describes the structure of 2,3-dihydro-1,3-benzothiazolo-2-selone, which was obtained by a reaction of 2-chlorobenzothiazole with sodium hydroselenide. It should be noted that, before us, the attempt to prepare this compound by the same reaction was unsuccessful (Shibata & Mitsunobu, 1992). Moreover, the preparation method of the title compound by the reaction of *o*-aminobenzothiol with CSe_2_ was previously known, but conclusive evidences were not provided (Warner, 1963).

The molecule of C_7_H_5_NSSe, I, is practically planar (r.m.s. deviation = 0.018Å) due to the presence of the long chain of conjugated bonds (Se1═C2–N3–C3A═C4–C5═C6–C7═C7A, Fig. 1). The geometrical parameters of I correspond well to those of the closer analog of I - *N*-methylbenzothiazole-2(3*H*)-selone (Husebye *et al.*, 1997) demonstrating that the sulfur atom does not take a significant part in the electron delocalization within the molecule.

In the crystal, the molecules of I form centrosymmetrical dimers by the intermolecular N3–H3···Se1^i^ hydrogen bonds (Fig. 2, Table 1). It is interesting to point out that the dimers have non-planar *ladder*-like structure (Fig. 3). The dimers are further linked into ribbons propagating in [0 1 0] by the weak attractive intermolecular Se1···S1^ii^ [Se···S distances are 3.7593 (4)Å] interactions (Nakanishi *et al.*, 2008) (Fig. 3). Symmetry codes: (i) -*x*+2, -*y*, -*z*+1; (ii) *x*, -1+*y*, *z*.

### Experimental

To a suspension of selenium (1.92 g, 24.3 mmol) in water (15 ml) was added a solution of NaBH_4_ (1.93 g, 50.8 mmol) in water (15 ml) with stirring at room temperature under argon. After 10 min, 2-chlorobenzothiazole (2.6 ml, 20 mmol) was added. The mixture was heated 3 h at 353 K and cooled to room temperature. Then to the solution was added 1*M* H_2_SO_4_ (20 ml) to give yellow precipitate (Figure 4). The crystalline powder was separated by filtration, washed with water and dried on air at 413 K. The solid was recrystallized from CH_2_Cl_2_ to give the selone as pale-yellow prisms. Yield is 73%. M.P. = 346-347 K. IR (KBr), ν (cm^-1^): 3431, 3010, 2343, 1595, 1490, 1456, 1421, 1319, 1244, 983, 750, 669; ^1^H NMR (DMSO-d_6_, 600 MHz, 303 K): δ = 7.34 (t, 1H, H6, J = 7.3), 7.41 (t, 1H, H5, J = 7.3), 7.47 (d, 1H, H7, J = 7.3), 7.75 (d, 1H, H4, J = 7.3), 14.40 (s, 1H, H3). Anal. Calcd. for C_7_H_5_NSSe: C, 39.27; H, 2.35; N, 6.54. Found: C, 39.18; H, 2.30; N, 6.47.

### Refinement

The amino hydrogen atom was localized in the difference Fourier map and included in the refinement with fixed positional and isotropic displacement parameters *U*_iso_(H) = 1.2*U*_eq_(N). The other hydrogen atoms were placed in calculated positions with C–H = 0.95Å and refined in the riding model with fixed isotropic displacement parameters *U*_iso_(H) = 1.2*U*_eq_(C).

### Crystal data

**Table:**

C_7_H_5_NSSe	*F*(000) = 416
*M**_r_* = 214.15	*D*_x_ = 1.952 Mg m^−^^3^
Monoclinic, *P*2_1_/*n*	Melting point = 346–347 K
Hall symbol: -P 2yn	Mo *K*α radiation, λ = 0.71073 Å
*a* = 8.0420 (4) Å	Cell parameters from 6338 reflections
*b* = 6.0818 (3) Å	θ = 2.6–30.0°
*c* = 15.1836 (7) Å	µ = 5.35 mm^−^^1^
β = 101.195 (1)°	*T* = 100 K
*V* = 728.50 (6) Å^3^	Prism, pale-yellow
*Z* = 4	0.30 × 0.21 × 0.18 mm

### Data collection

**Table:**

Bruker SMART 1K CCD diffractometer	2108 independent reflections
Radiation source: fine-focus sealed tube	2042 reflections with *I* > 2σ(*I*)
graphite	*R*_int_ = 0.020
φ and ω scans	θ_max_ = 30.0°, θ_min_ = 2.7°
Absorption correction: multi-scan (*SADABS*; Sheldrick, 1998)	*h* = −11→11
*T*_min_ = 0.297, *T*_max_ = 0.446	*k* = −8→8
8129 measured reflections	*l* = −20→21

### Refinement

**Table:**

Refinement on *F*^2^	Primary atom site location: structure-invariant direct methods
Least-squares matrix: full	Secondary atom site location: difference Fourier map
*R*[*F*^2^ > 2σ(*F*^2^)] = 0.016	Hydrogen site location: difference Fourier map
*wR*(*F*^2^) = 0.041	H-atom parameters constrained
*S* = 1.00	*w* = 1/[σ^2^(*F*_o_^2^) + (0.023*P*)^2^ + 0.371*P*] where *P* = (*F*_o_^2^ + 2*F*_c_^2^)/3
2108 reflections	(Δ/σ)_max_ = 0.001
91 parameters	Δρ_max_ = 0.44 e Å^−^^3^
0 restraints	Δρ_min_ = −0.29 e Å^−^^3^

### Special details

**Table:**

Geometry. All s.u.'s (except the s.u. in the dihedral angle between two l.s. planes) are estimated using the full covariance matrix. The cell s.u.'s are taken into account individually in the estimation of s.u.'s in distances, angles and torsion angles; correlations between s.u.'s in cell parameters are only used when they are defined by crystal symmetry. An approximate (isotropic) treatment of cell s.u.'s is used for estimating s.u.'s involving l.s. planes.
Refinement. Refinement of *F*^2^ against ALL reflections. The weighted *R*-factor *wR* and goodness of fit *S* are based on *F*^2^, conventional *R*-factors *R* are based on *F*, with *F* set to zero for negative *F*^2^. The threshold expression of *F*^2^ > σ*F*^2^ is used only for calculating *R*-factors(gt) *etc*. and is not relevant to the choice of reflections for refinement. *R*-factors based on *F*^2^ are statistically about twice as large as those based on *F*, and *R*-factors based on ALL data will be even larger.

### Fractional atomic coordinates and isotropic or equivalent isotropic displacement parameters (Å^2^)

**Table:**

	*x*	*y*	*z*	*U*_iso_*/*U*_eq_	
Se1	1.277060 (14)	0.076249 (19)	0.579287 (8)	0.01478 (5)	
S1	1.17970 (3)	0.51668 (5)	0.661496 (19)	0.01522 (6)	
C2	1.12217 (14)	0.27701 (18)	0.60187 (7)	0.01301 (19)	
N3	0.95281 (12)	0.26504 (16)	0.57732 (6)	0.01391 (18)	
H3	0.9022	0.1543	0.5457	0.017*	
C3A	0.86204 (14)	0.43853 (18)	0.60496 (8)	0.0126 (2)	
C4	0.68634 (15)	0.4594 (2)	0.59126 (8)	0.0155 (2)	
H4	0.6135	0.3500	0.5601	0.019*	
C5	0.62189 (15)	0.6465 (2)	0.62499 (8)	0.0170 (2)	
H5	0.5026	0.6659	0.6160	0.020*	
C6	0.72815 (15)	0.8072 (2)	0.67190 (8)	0.0167 (2)	
H6	0.6801	0.9335	0.6940	0.020*	
C7	0.90347 (15)	0.7839 (2)	0.68649 (8)	0.0160 (2)	
H7	0.9762	0.8923	0.7185	0.019*	
C7A	0.96916 (14)	0.59737 (19)	0.65275 (8)	0.0133 (2)	

### Atomic displacement parameters (Å^2^)

**Table:**

	*U*^11^	*U*^22^	*U*^33^	*U*^12^	*U*^13^	*U*^23^
Se1	0.01251 (7)	0.01418 (7)	0.01708 (7)	0.00142 (4)	0.00145 (4)	−0.00172 (4)
S1	0.01046 (12)	0.01552 (13)	0.01887 (13)	−0.00113 (9)	0.00088 (10)	−0.00449 (10)
C2	0.0133 (5)	0.0130 (5)	0.0125 (5)	0.0000 (4)	0.0020 (4)	0.0006 (4)
N3	0.0125 (4)	0.0132 (4)	0.0155 (4)	−0.0010 (3)	0.0015 (3)	−0.0021 (3)
C3A	0.0121 (5)	0.0134 (5)	0.0123 (5)	−0.0008 (4)	0.0022 (4)	0.0001 (4)
C4	0.0124 (5)	0.0177 (5)	0.0158 (5)	−0.0016 (4)	0.0013 (4)	0.0001 (4)
C5	0.0128 (5)	0.0219 (6)	0.0168 (5)	0.0018 (4)	0.0040 (4)	0.0019 (4)
C6	0.0172 (5)	0.0179 (5)	0.0159 (5)	0.0032 (4)	0.0052 (4)	0.0001 (4)
C7	0.0160 (5)	0.0163 (5)	0.0154 (5)	0.0000 (4)	0.0024 (4)	−0.0026 (4)
C7A	0.0114 (5)	0.0149 (5)	0.0134 (5)	−0.0012 (4)	0.0021 (4)	−0.0009 (4)

### Geometric parameters (Å, °)

**Table:**

Se1—C2	1.8236 (11)	C4—C5	1.3890 (17)
S1—C2	1.7308 (12)	C4—H4	0.9500
S1—C7A	1.7430 (12)	C5—C6	1.3986 (17)
C2—N3	1.3425 (14)	C5—H5	0.9500
N3—C3A	1.3933 (14)	C6—C7	1.3913 (16)
N3—H3	0.8800	C6—H6	0.9500
C3A—C4	1.3935 (16)	C7—C7A	1.3906 (16)
C3A—C7A	1.3997 (15)	C7—H7	0.9500
			
C2—S1—C7A	92.31 (5)	C4—C5—C6	121.69 (11)
N3—C2—S1	110.16 (8)	C4—C5—H5	119.2
N3—C2—Se1	127.21 (9)	C6—C5—H5	119.2
S1—C2—Se1	122.63 (6)	C7—C6—C5	120.67 (11)
C2—N3—C3A	115.97 (10)	C7—C6—H6	119.7
C2—N3—H3	122.0	C5—C6—H6	119.7
C3A—N3—H3	122.0	C7A—C7—C6	118.04 (11)
N3—C3A—C4	126.82 (10)	C7A—C7—H7	121.0
N3—C3A—C7A	111.91 (10)	C6—C7—H7	121.0
C4—C3A—C7A	121.25 (10)	C7—C7A—C3A	120.96 (11)
C5—C4—C3A	117.37 (11)	C7—C7A—S1	129.39 (9)
C5—C4—H4	121.3	C3A—C7A—S1	109.64 (8)
C3A—C4—H4	121.3		
			
C7A—S1—C2—N3	0.42 (9)	C5—C6—C7—C7A	0.29 (18)
C7A—S1—C2—Se1	−179.05 (7)	C6—C7—C7A—C3A	0.46 (17)
S1—C2—N3—C3A	−1.00 (13)	C6—C7—C7A—S1	−178.39 (9)
Se1—C2—N3—C3A	178.44 (8)	N3—C3A—C7A—C7	−179.85 (10)
C2—N3—C3A—C4	−177.14 (11)	C4—C3A—C7A—C7	−1.41 (17)
C2—N3—C3A—C7A	1.19 (14)	N3—C3A—C7A—S1	−0.80 (12)
N3—C3A—C4—C5	179.72 (11)	C4—C3A—C7A—S1	177.64 (9)
C7A—C3A—C4—C5	1.53 (17)	C2—S1—C7A—C7	179.17 (12)
C3A—C4—C5—C6	−0.77 (18)	C2—S1—C7A—C3A	0.22 (9)
C4—C5—C6—C7	−0.13 (18)		

### Hydrogen-bond geometry (Å, °)

**Table:**

*D*—H···*A*	*D*—H	H···*A*	*D*···*A*	*D*—H···*A*
N3—H3···Se1^i^	0.88	2.56	3.4165 (10)	163

Symmetry codes: (i) −*x*+2, −*y*, −*z*+1.
